# Supplementary material for: Methionine metabolism influences genomic architecture and gene expression through H3K4me3 peak width
Source: Nat Commun. 2018 May 16;9:1955. doi: 10.1038/s41467-018-04426-y (PMC5955993; doi:10.1038/s41467-018-04426-y)
Supplement: Supplementary file 1 — Supplementary Information [file 41467_2018_4426_MOESM1_ESM.pdf]

Methionine metabolism influences genomic architecture and gene expression through H3K4me3 peak width

Dai *et al.*

Supplementary Figures

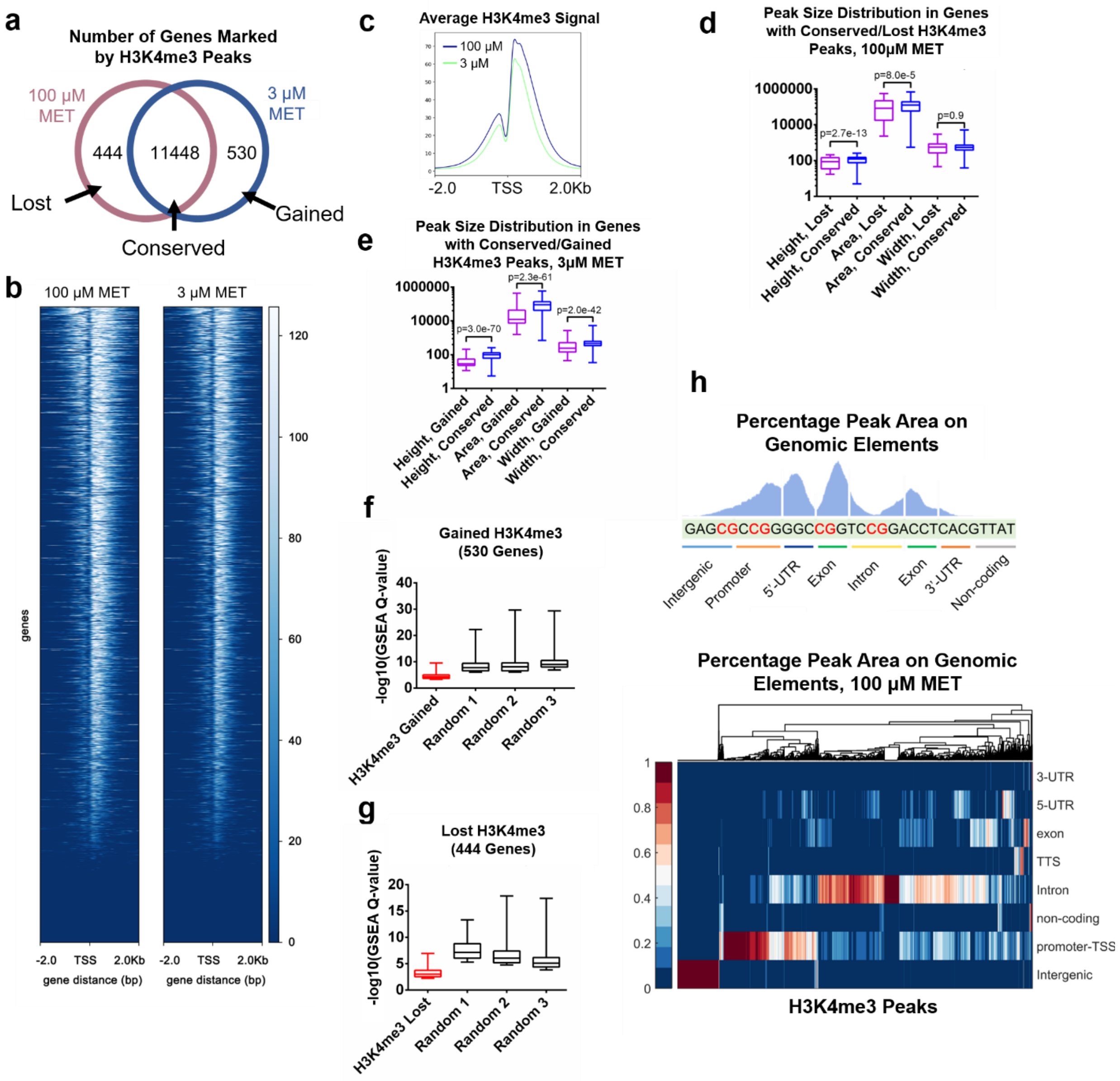

Supplementary Figure 1 (Related to Fig. 1). MR reduces H3K4me3 but maintains its genomic distribution in human cancer cells.

a. Number of genes marked by H3K4me3 peaks under high and low methionine conditions in human cancer cells.

b. Average H3K4me3 profiles around transcription start sites (TSS)s under high and low methionine conditions in human cancer cells.

c. Heatmap showing H3K4me3 signal around TSS of each marked gene under high and low methionine conditions in human cancer cells.
d. Distribution of peak height, area and width under high methionine condition in genes with conserved or lost H3K4me3 peaks in human cancer cells. The P-values were computed from the Wilcoxon rank-sum test. Box limits are the 25th and 75th percentiles, center lines are medians, and the whiskers are the minimal and maximal values.
e. Distribution of peak height, area and width under low methionine condition in genes with conserved or gained H3K4me3 peaks in human cancer cells. The P-values were computed from the Wilcoxon rank-sum test. Box limits are the 25th and 75th percentiles, center lines are medians, and the whiskers are the minimal and maximal values.
f. Distributions of pathway enrichment Q-values in 530 genes with gained H3K4me3 and random gene sets with identical size in human cancer cells. Box limits are the 25th and 75th percentiles, center lines are medians, and the whiskers are the minimal and maximal values.
g. Same as in (f) but for 444 genes with loss of H3K4me3.
h. Definition of percentage peak area on genomic elements and its distribution under high methionine condition in human cancer cells.

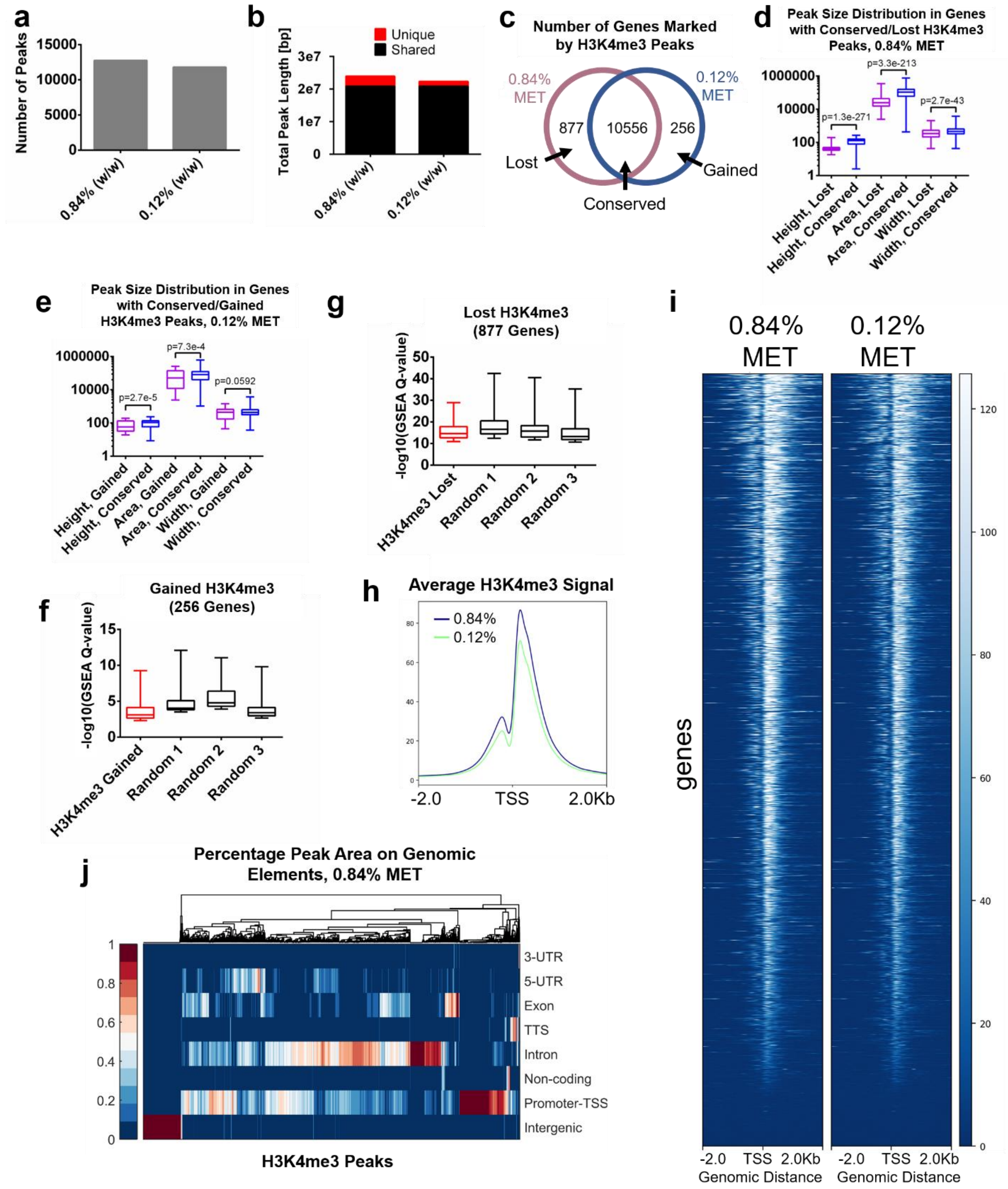

Supplementary Figure 2 (Related to Fig. 1). MR reduces H3K4me3 but maintains its genomic distribution in mouse liver.

a. Shared and unique length of peak regions under high and low methionine conditions in mouse liver.
b. Number of peaks called under high and low methionine conditions in mouse liver.
c. Number of genes marked by H3K4me3 peaks under high and low methionine conditions in mouse liver.
d. Distribution of peak height, area and width under high methionine condition in genes with conserved or lost H3K4me3 peaks in mouse liver. The P-values were computed from the Wilcoxon rank-sum test. Box limits are the 25th and 75th percentiles, center lines are medians, and the whiskers are the minimal and maximal values.
e. Distribution of peak height, area and width under low methionine condition in genes with conserved or gained H3K4me3 peaks in mouse liver. The P-values were computed from the Wilcoxon rank-sum test. Box limits are the 25th and 75th percentiles, center lines are medians, and the whiskers are the minimal and maximal values.
f. Distribution of pathway enrichment Q-values in 256 genes with gained H3K4me3 and random gene sets with identical size in mouse liver. Box limits are the 25th and 75th percentiles, center lines are medians, and the whiskers are the minimal and maximal values.
g. Same as in (f) but for 877 genes with loss of H3K4me3.
h. Average H3K4me3 profiles around TSSs under high and low methionine conditions in mouse liver.
i. Heatmap showing H3K4me3 signal around TSS of each marked gene under high and low methionine conditions in mouse liver.
j. Percentage peak area on genomic elements for each H3K4me3 peak under high methionine condition in mouse liver.

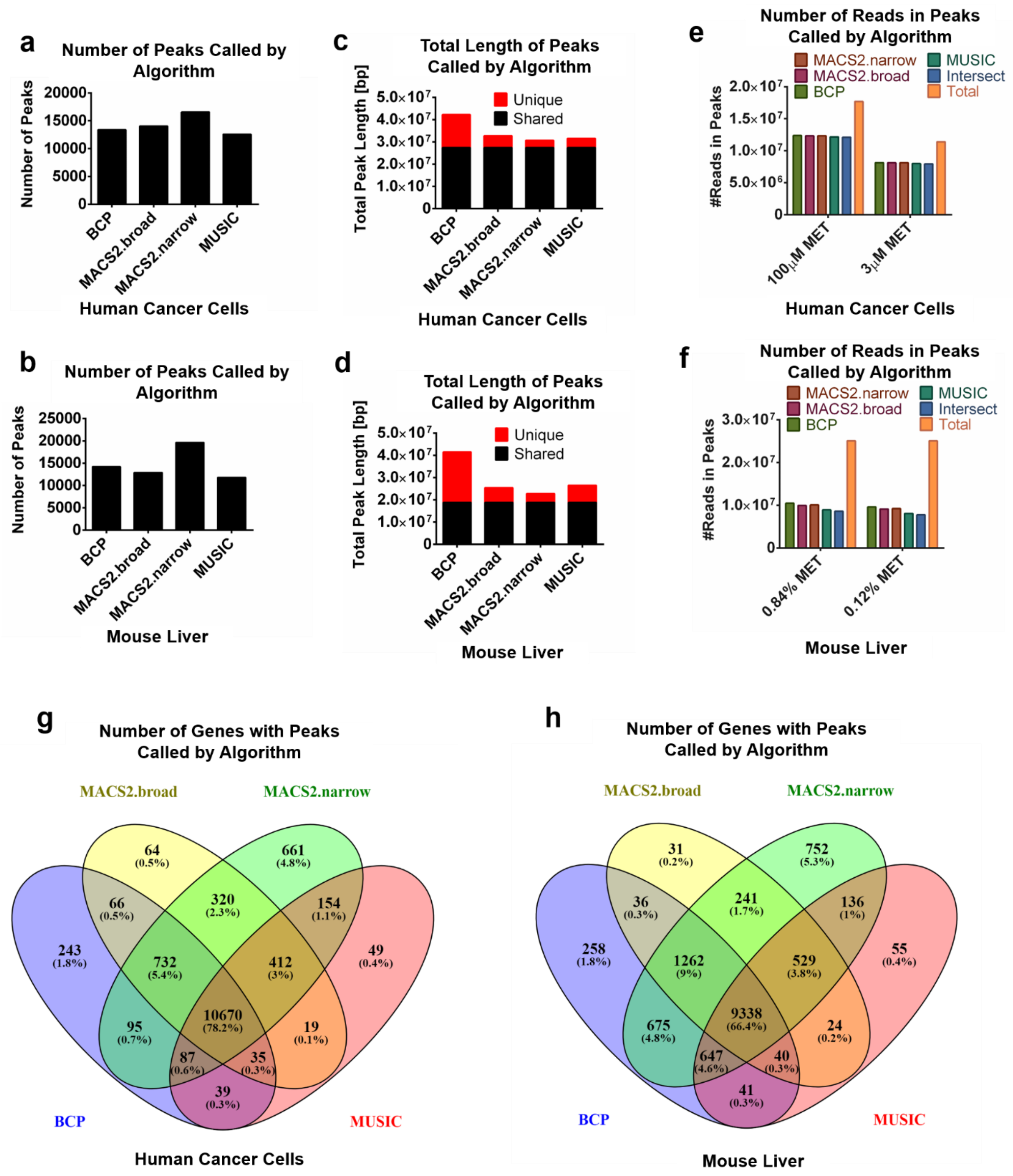

Supplementary Figure 3 (Related to Fig. 1). Genomic location of H3K4me3 peaks is consistent under different ChIP-seq peak calling algorithms.

a. Number of peaks called by different peak-calling algorithms in human cancer cells.

b. Same as in (a) but for mouse liver.

c. Total length of peaks called by different peak-calling algorithms in human cancer cells.

d. Same as in (c) but for mouse liver.

e. Number of reads in peaks called by different peak-calling algorithms in human cancer cells.

f. Same as in (e) but for mouse liver.

g. Venn diagram of genes marked by peaks called by different peak-calling algorithms in human cancer cells.

h. Same as in (g) but for mouse liver.

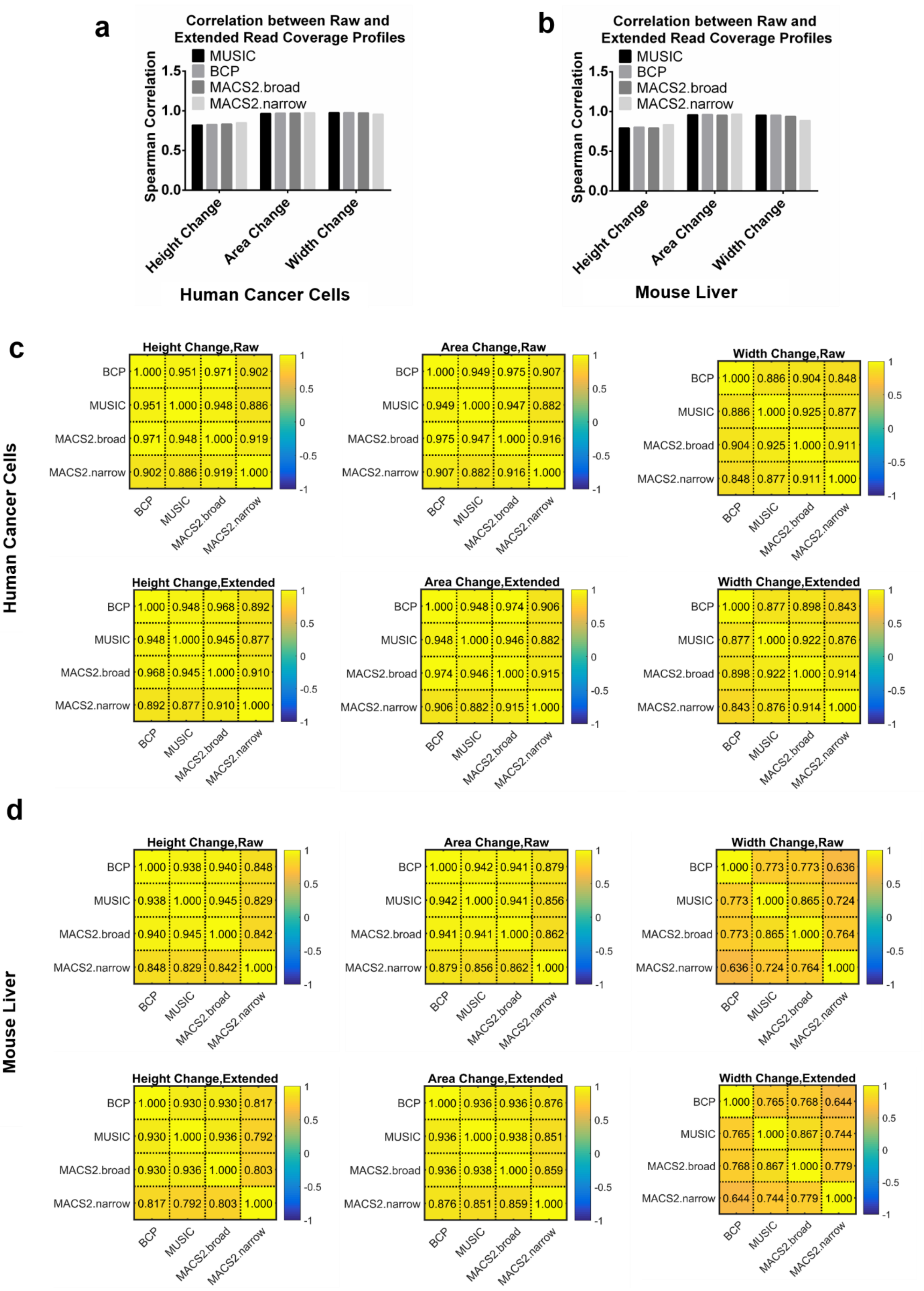
a
Correlation between Raw and Extended Read Coverage Profiles
MUSIC
BCP
MACS2.broad
MACS2.narrow
Spearman Correlation
1.5
1.0
0.5
0.0
Height Change
Area Change
Width Change
Human Cancer Cells
b
Correlation between Raw and Extended Read Coverage Profiles
MUSIC
BCP
MACS2.broad
MACS2.narrow
Spearman Correlation
1.5
1.0
0.5
0.0
Height Change
Area Change
Width Change
Mouse Liver
c
Human Cancer Cells
Height Change,Raw
BCP 1.000 0.951 0.971 0.902
MUSIC 0.951 1.000 0.948 0.886
MACS2.broad 0.971 0.948 1.000 0.919
MACS2.narrow 0.902 0.886 0.919 1.000
Area Change,Raw
BCP 1.000 0.949 0.975 0.907
MUSIC 0.949 1.000 0.947 0.882
MACS2.broad 0.975 0.947 1.000 0.916
MACS2.narrow 0.907 0.882 0.916 1.000
Width Change,Raw
BCP 1.000 0.886 0.904 0.848
MUSIC 0.886 1.000 0.925 0.877
MACS2.broad 0.904 0.925 1.000 0.911
MACS2.narrow 0.848 0.877 0.911 1.000
Height Change,Extended
BCP 1.000 0.948 0.968 0.892
MUSIC 0.948 1.000 0.945 0.877
MACS2.broad 0.968 0.945 1.000 0.910
MACS2.narrow 0.892 0.877 0.910 1.000
Area Change,Extended
BCP 1.000 0.948 0.974 0.906
MUSIC 0.948 1.000 0.946 0.882
MACS2.broad 0.974 0.946 1.000 0.915
MACS2.narrow 0.906 0.882 0.915 1.000
Width Change,Extended
BCP 1.000 0.877 0.898 0.843
MUSIC 0.877 1.000 0.922 0.876
MACS2.broad 0.898 0.922 1.000 0.914
MACS2.narrow 0.843 0.876 0.914 1.000
d
Mouse Liver
Height Change,Raw
BCP 1.000 0.938 0.940 0.848
MUSIC 0.938 1.000 0.945 0.829
MACS2.broad 0.940 0.945 1.000 0.842
MACS2.narrow 0.848 0.829 0.842 1.000
Area Change,Raw
BCP 1.000 0.942 0.941 0.879
MUSIC 0.942 1.000 0.941 0.856
MACS2.broad 0.941 0.941 1.000 0.862
MACS2.narrow 0.879 0.856 0.862 1.000
Width Change,Raw
BCP 1.000 0.773 0.773 0.636
MUSIC 0.773 1.000 0.865 0.724
MACS2.broad 0.773 0.865 1.000 0.764
MACS2.narrow 0.636 0.724 0.764 1.000
Height Change,Extended
BCP 1.000 0.930 0.930 0.817
MUSIC 0.930 1.000 0.936 0.792
MACS2.broad 0.930 0.936 1.000 0.803
MACS2.narrow 0.817 0.792 0.803 1.000
Area Change,Extended
BCP 1.000 0.936 0.936 0.876
MUSIC 0.936 1.000 0.938 0.851
MACS2.broad 0.936 0.938 1.000 0.859
MACS2.narrow 0.876 0.851 0.859 1.000
Width Change,Extended
BCP 1.000 0.765 0.768 0.644
MUSIC 0.765 1.000 0.867 0.744
MACS2.broad 0.768 0.867 1.000 0.779
MACS2.narrow 0.644 0.744 0.779 1.000
BCP MUSIC MACS2.broad MACS2.narrow
1 0.5 0 -0.5 -1

Supplementary Figure 4 (Related to Fig. 1). Quantification of H3K4me3 peak dynamics is consistent under different ChIP-seq data analysis pipelines.

a. Spearman correlation between raw (i.e. without extension of reads to the whole fragment) and extended (i.e. with extension of reads to the whole fragment) read coverage profiles for changes in height, area and width in human cancer cells.
b. Same as in (a) but for mouse liver.
c. Spearman correlation between peak calling algorithms for changes in height, area and width in human cancer cells.
d. Same as in (c) but for mouse liver.

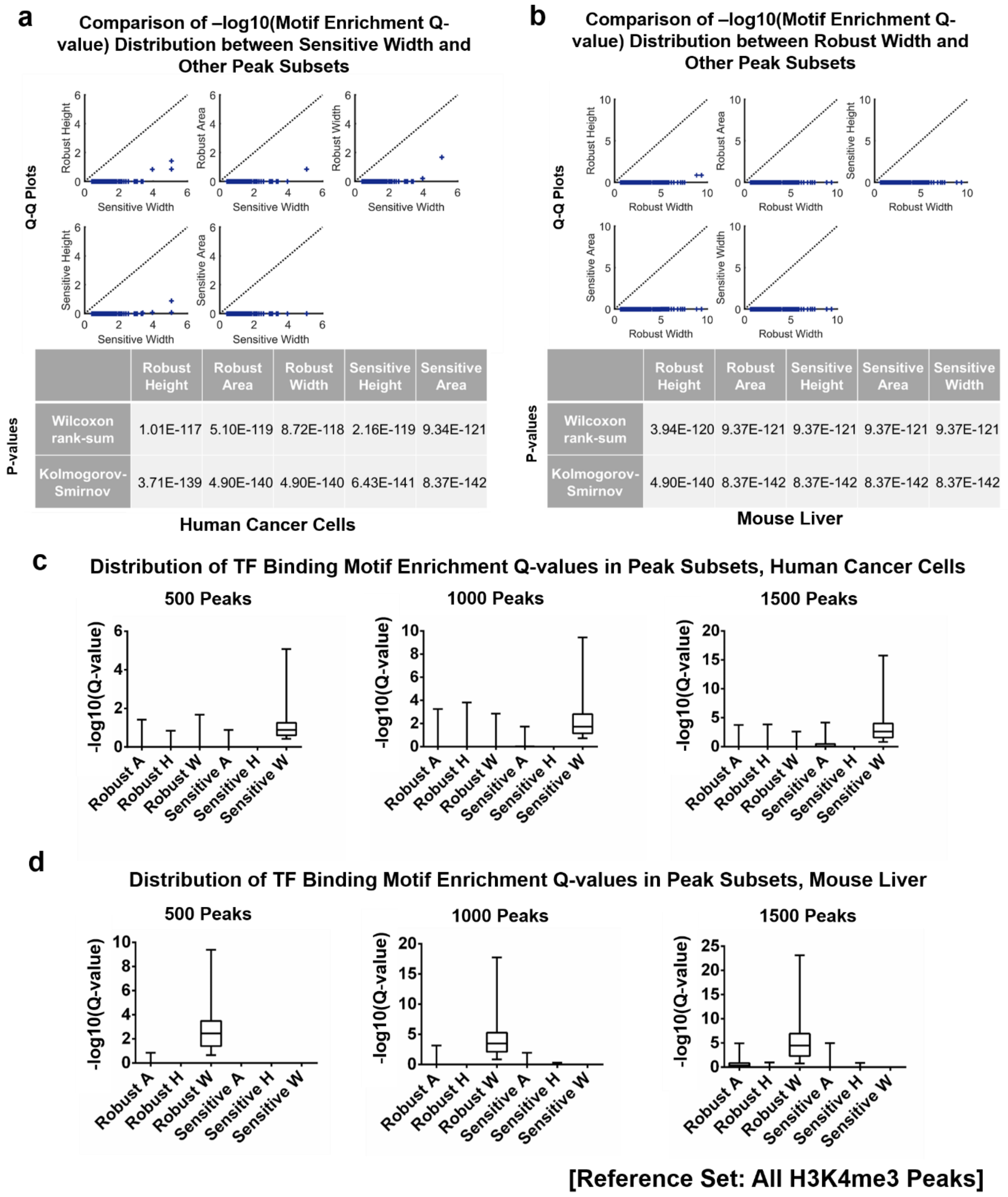

a — Human Cancer Cells (P-values)

	Robust Height	Robust Area	Robust Width	Sensitive Height	Sensitive Area
Wilcoxon rank-sum	1.01E-117	5.10E-119	8.72E-118	2.16E-119	9.34E-121
Kolmogorov-Smirnov	3.71E-139	4.90E-140	4.90E-140	6.43E-141	8.37E-142

b — Mouse Liver (P-values)

	Robust Height	Robust Area	Sensitive Height	Sensitive Area	Sensitive Width
Wilcoxon rank-sum	3.94E-120	9.37E-121	9.37E-121	9.37E-121	9.37E-121
Kolmogorov-Smirnov	4.90E-140	8.37E-142	8.37E-142	8.37E-142	8.37E-142

Supplementary Figure 5 (Related to Fig. 3). H3K4me3 width dynamics encode cell type specific TF binding.

a. Quantile-Quantile (Q-Q) plots, Wilcoxon rank-sum P-values and Kolmogorov-Smirnov P-values comparing distributions of TF binding motif enrichment Q-values in other peak subsets to the peak subset with sensitive width in human cancer cells. Each peak subset contains 500 peaks.
b. Same as in (a) but for robust width in mouse liver.
c. TF binding motif enrichment Q-value distributions in 500, 1000 and 1500 H3K4me3 peaks with different dynamics under MR in human cancer cells. Box limits are the 25th and 75th percentiles, center lines are medians, and the whiskers are the minimal and maximal values.
d. Same as in (c) but for mouse liver.

a TFs predicted to associate with H3K4me3 peak width

System	TF	Accession Numbers
Human Cancer Cells (Sensitive Width)	MYC	• GEO:GSM2576763
Human Cancer Cells (Sensitive Width)	ELF1	• GEO:GSM1010765
Mouse Liver (Robust Width)	HNF4a	• ArrayExpress:E-TABM-722
Mouse Liver (Robust Width)	RXRa	• GEO:GSM541300,GSM541301,GSM541304,GSM541305 (Dataset 1); • GEO:GSM1138337 (Dataset 2); • GEO:GSM1299600 (Dataset 3)

b TFs predicted not to associate with H3K4me3 peak width

System	TF	Accession Numbers
Human Cancer Cells	CTCF	• GEO:GSM1010903
Mouse Liver	E2F4	• GEO:GSM2040953, GSM2040954

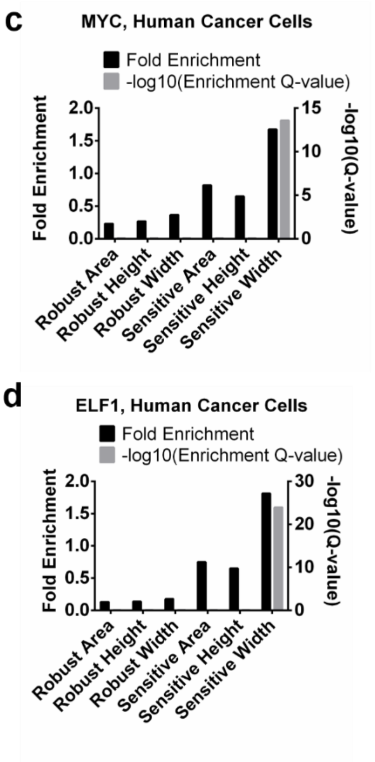

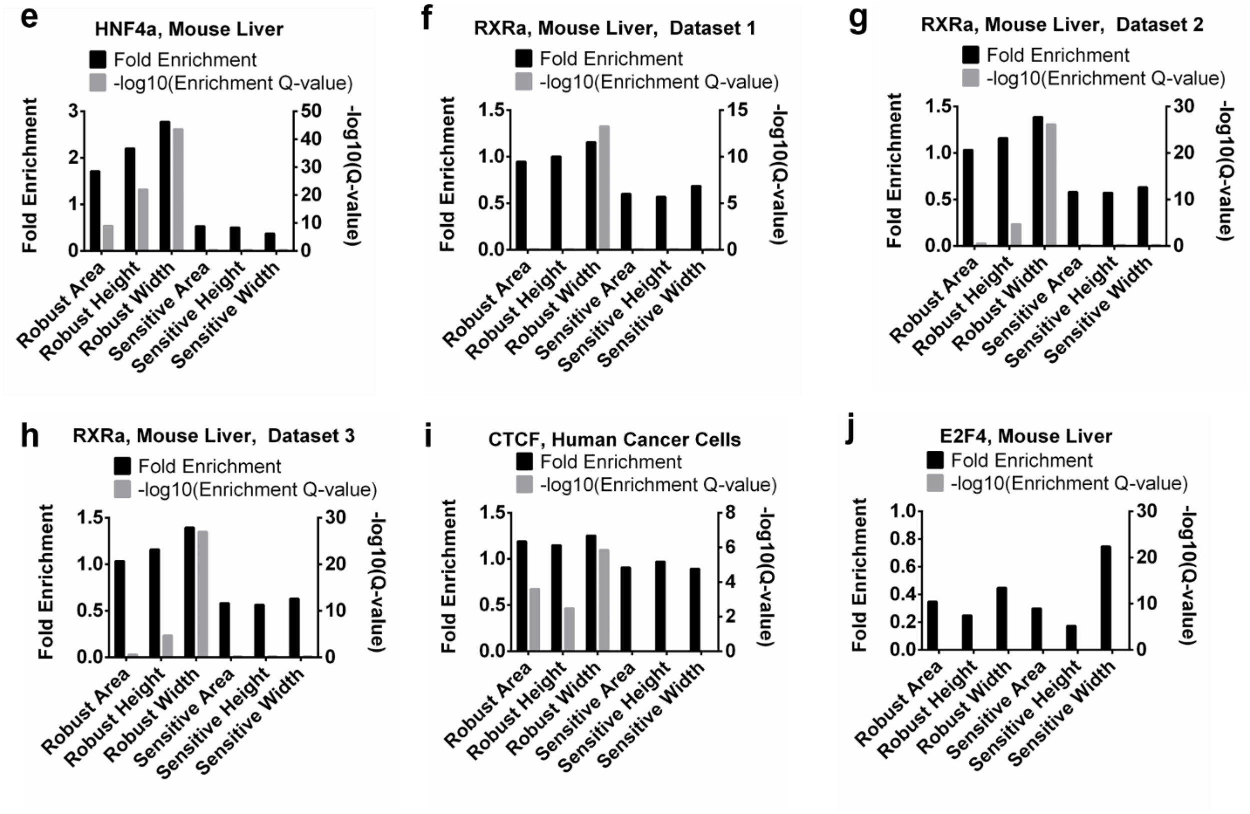

Supplementary Figure 6 (Related to Fig. 3). Validation of TFs associated with H3K4me3 width dynamics.

a. Table of ChIP-seq datasets used in the analysis of Transcription Factors (TF)s putatively associated with H3K4me3 peak width. Column 1 represents the system studies, Column 2 denotes the TF used, and Column 3 defines the accession numbers for the ChIP-seq data.
b. Table of ChIP-seq datasets as in (a) but for TFs putatively not associated with H3K4me3 peak width.
c. Fraction of peaks bound by MYC in a subset of peaks (defined on the x-axis) relative to the fraction of peaks bound by the TF in all H3K4me3 peaks (i.e. fold enrichment) in human cancer cells. Q-values represent an adjusted (Benjamin-Hochberg method) P-value obtained from a one-side Fisher's exact test. Further information is contained in the Methods.
d. Same as in (c) but for ELF1 in human cancer cells.
e. Same as in (c) but for HNF4a in mouse liver.
f. Same as in (c) but for RXRa in mouse liver based on Dataset 1.
g. Same as in (c) but for RXRa in mouse liver based on Dataset 2.
h. Same as in (c) but for RXRa in mouse liver based on Dataset 3.
i. Same as in (c) but for CTCF in human cancer cells.
j. Same as in (c) but for E2F4 in mouse liver.

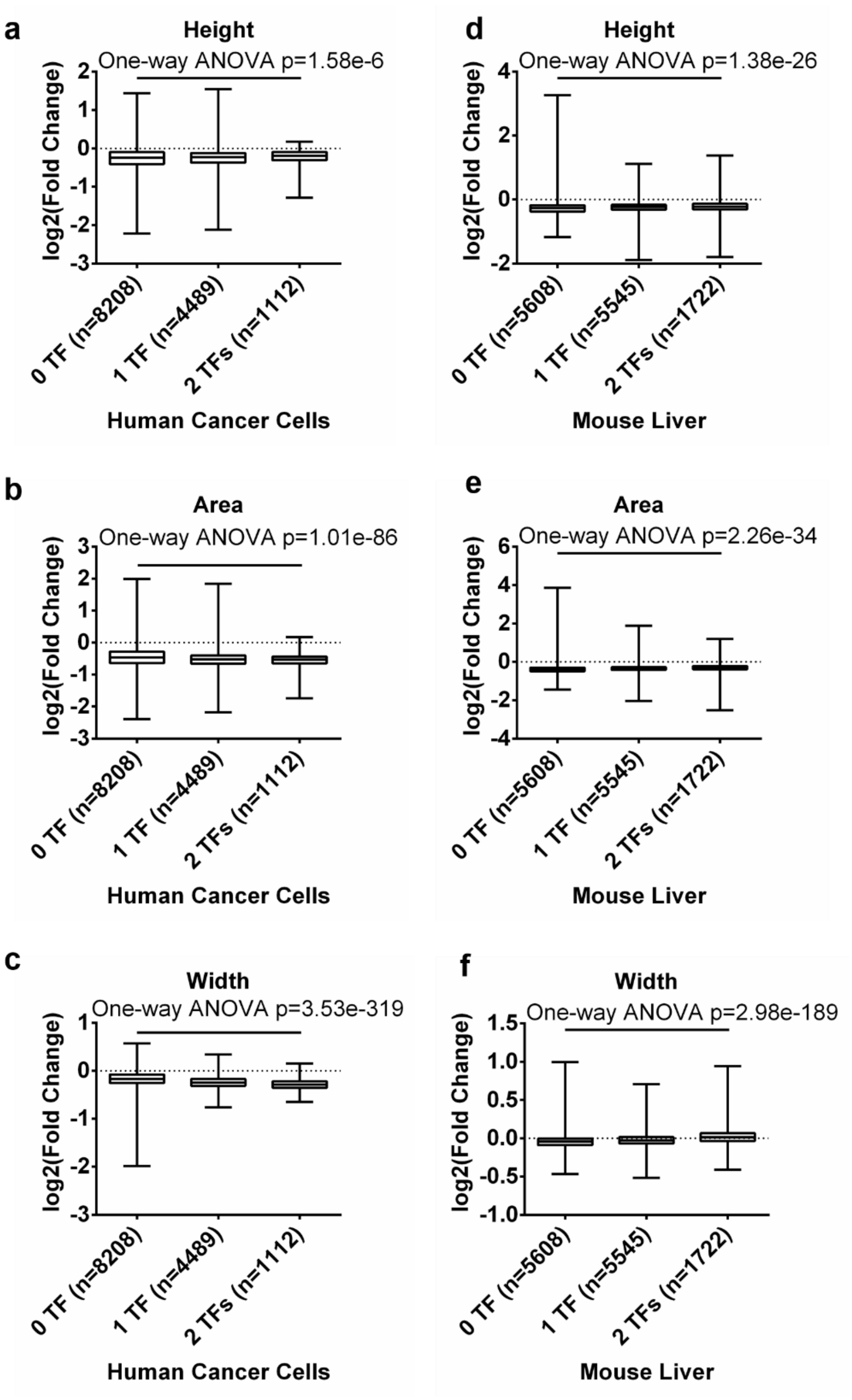

Supplementary Figure 7. (Related to Fig. 3). Cell type specific TF binding associates with H3K4me3 width dynamics.

a. Distributions of H3K4me3 height changes (high and low methionine) in peaks bound by 0,1 or 2 TFs putatively associated with H3K4me3 width in human cancer cells. Box limits are the 25th

and 75th percentiles, center lines are medians, and the whiskers are the minimal and maximal values.

b. Same as in (a) but for area.

c. Same as in (a) but for width.

d. Distribution of H3K4me3 height changes in peaks bound by 0, 1, or 2 TFs putatively associated with H3K4me3 width in mouse liver. Box limits are the 25th and 75th percentiles, center lines are medians, and the whiskers are the minimal and maximal values.

e. Same as in (d) but for area.

f. Same as in (d) but for width.

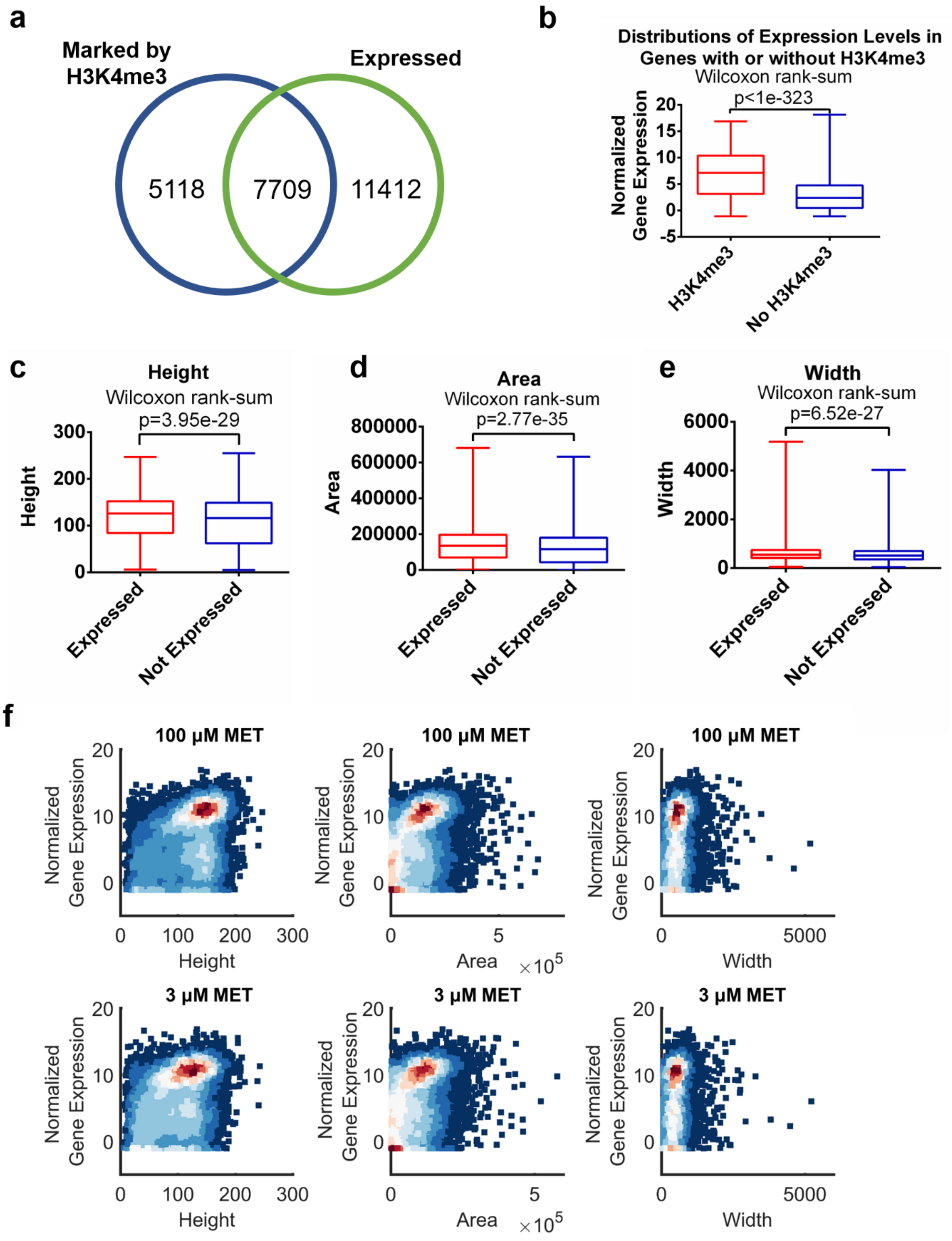

a
Marked by H3K4me3
Expressed
5118
7709
11412
b
Distributions of Expression Levels in Genes with or without H3K4me3
Wilcoxon rank-sum
p<1e-323
Normalized Gene Expression
H3K4me3
No H3K4me3
c
Height
Wilcoxon rank-sum
p=3.95e-29
Expressed
Not Expressed
d
Area
Wilcoxon rank-sum
p=2.77e-35
e
Width
Wilcoxon rank-sum
p=6.52e-27
f
100 μM MET
3 μM MET
Normalized Gene Expression
Height
Area
$\times 10^5$
Width

Supplementary Figure 8 (Related to Fig. 4). Correlation between H3K4me3 and gene expression in human cancer cells.

a. Number of genes marked by H3K4me3 peaks, expressed genes and their overlap in human cancer cells.
b. Distribution of expression levels in genes with or without H3K4me3 in human cancer cells under high methionine conditions. Box limits are the 25th and 75th percentiles, center lines are medians, and the whiskers are the minimal and maximal values.
c. Distribution of height in H3K4me3 peaks associated with expressed or non-expressed genes in human cancer cells under high methionine conditions. Box limits are the 25th and 75th percentiles, center lines are medians, and the whiskers are the minimal and maximal values.
d. Same as in (c) but for area.
e. Same as in (c) but for width.
f. Density scatter plots comparing peak heights, areas and widths with gene expression levels under high and low methionine conditions in human cancer cells.

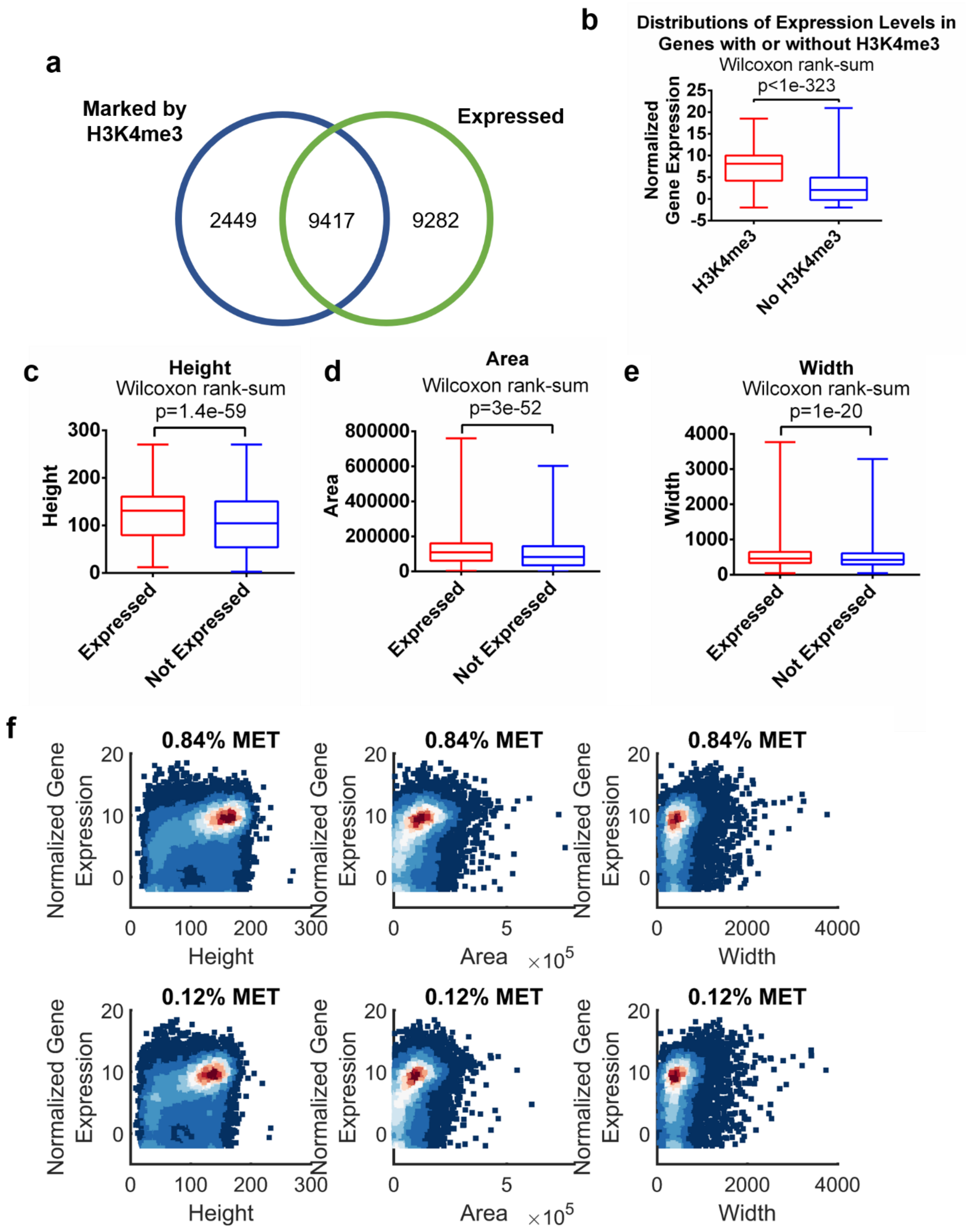

Supplementary Figure 9 (Related to Fig. 4). Correlation between H3K4me3 and gene expression in mouse liver.

a. Number of genes marked by H3K4me3 peaks, expressed genes and their overlap in mouse liver.
b. Distribution of expression levels in genes with or without H3K4me3 in mouse liver under high methionine conditions. Box limits are the 25th and 75th percentiles, center lines are medians, and the whiskers are the minimal and maximal values.
c. Distribution of heights in H3K4me3 peaks associated with expressed or non-expressed genes in mouse liver under high methionine conditions. Box limits are the 25th and 75th percentiles, center lines are medians, and the whiskers are the minimal and maximal values.
d. Same as in (c) but for area.
e. Same as in (c) but for width.
f. Density scatter plots comparing peak heights, areas and widths with gene expression levels under high and low methionine conditions in mouse liver.

a

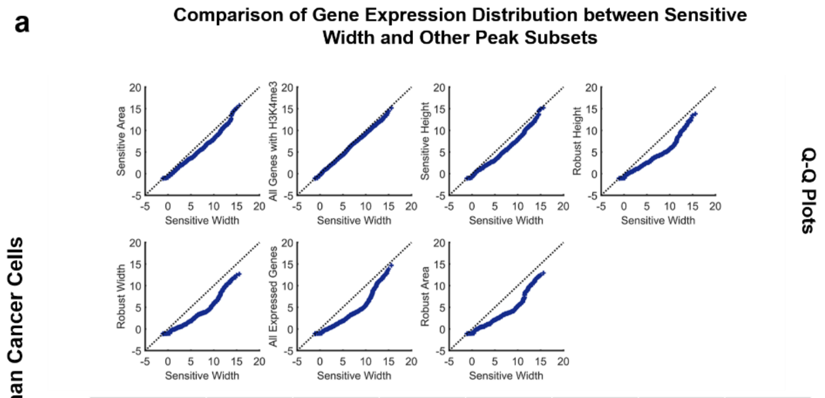

P-values

	Sensitive Area	All Genes with H3K4me3	Sensitive Height	Robust Height	Robust Width	All Expressed Genes	Robust Area
Wilcoxon rank-sum	6.30E-5	0.016	9.76E-6	1.17E-13	4.46E-15	1.81E-26	1.46E-16
Kolmogorov-Smirnov	2.58E-4	0.014	8.64E-5	6.50E-11	6.17E-10	2.65E-21	1.48E-12

b

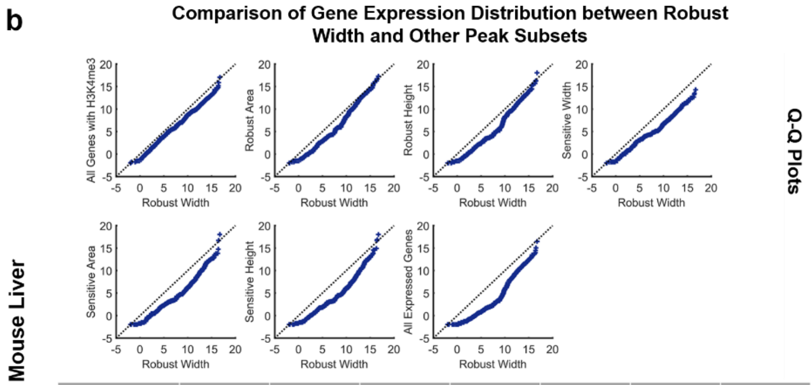

P-values

	All Genes with H3K4me3	Robust Area	Robust Height	Sensitive Width	Sensitive Area	Sensitive Height	All Expressed Genes
Wilcoxon rank-sum	3.70E-14	7.91E-07	1.05E-10	3.71E-24	5.96E-24	1.69E-24	9.94E-57
Kolmogorov-Smirnov	1.86E-15	2.54E-07	2.50E-08	1.50E-28	2.37E-25	2.85E-25	5.55E-43

Supplementary Figure 10 (Related to Fig. 4). Comparison of gene expression levels in different peak subsets.

a. Q-Q plots, Wilcoxon rank-sum P-values and Kolmogorov-Smirnov P-values comparing distributions of gene expression levels associated with other peak subsets to the peak subset with sensitive width in human cancer cells.

b. Same as in (a) but for robust width in mouse liver.

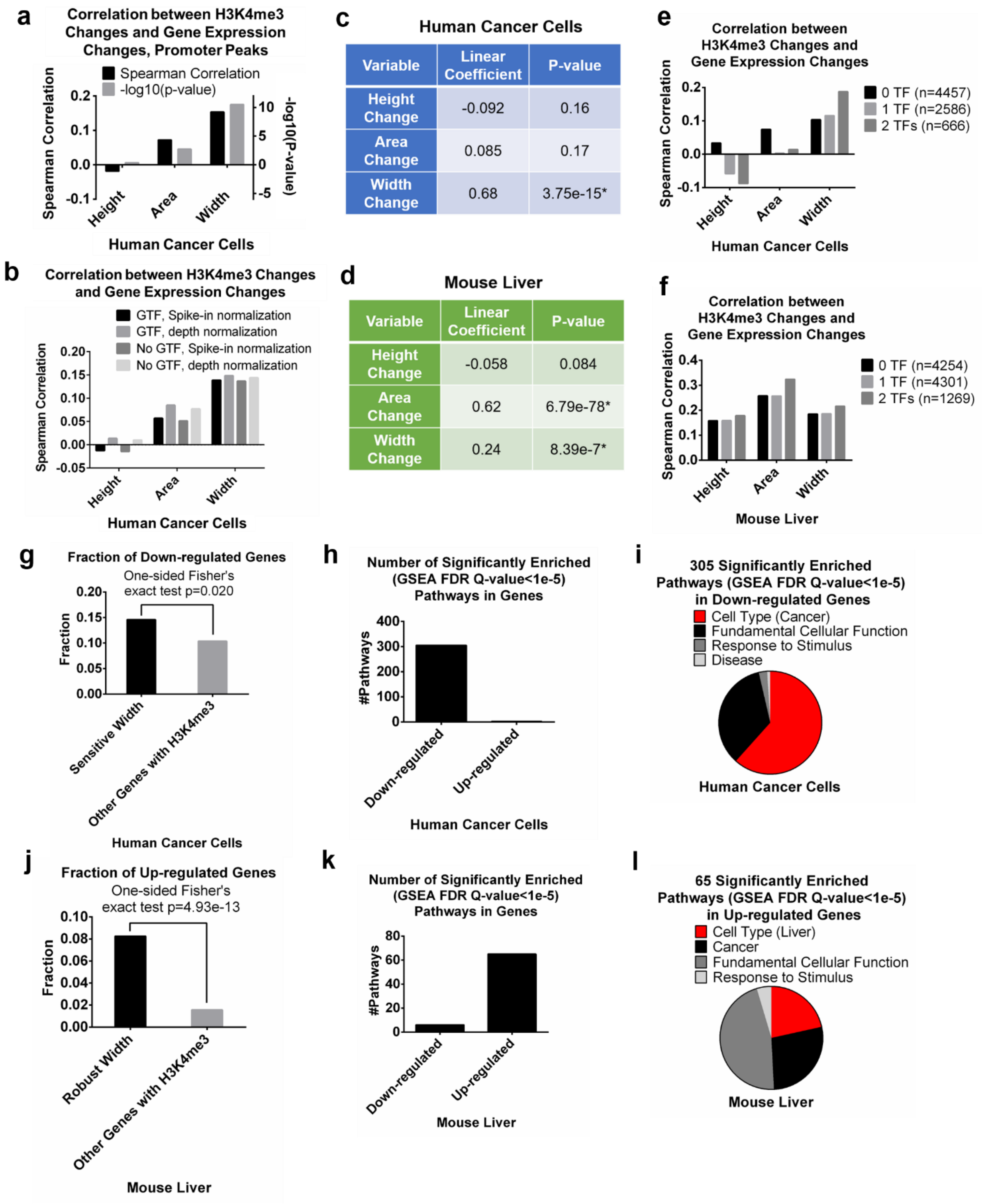

Variable	Linear Coefficient	P-value
Height Change	-0.092	0.16
Area Change	0.085	0.17
Width Change	0.68	3.75e-15*

Variable	Linear Coefficient	P-value
Height Change	-0.058	0.084
Area Change	0.62	6.79e-78*
Width Change	0.24	8.39e-7*

Supplementary Figure 11 (Related to Fig. 4). H3K4me3 width dynamics correlate with alterations in expression of cell type specific genes.

a. Spearman's rank correlation coefficients between H3K4me3 changes and gene expression changes with restriction of the analysis to promoter peaks in human cancer cells.
b. Spearman's rank correlation coefficients between H3K4me3 changes and gene expression changes using different methods for data analysis in human cancer cells.
c. Linear coefficients and P-values in the linear regression model predicting changes in gene expression from changes in height, area and width in human cancer cells.
d. Same as (c) but for mouse liver.
e. Spearman's rank correlation coefficients between H3K4me3 changes and gene expression changes in genes associated with H3K4me3 peaks bound by 0, 1, or 2 TFs putatively associated with H3K4me3 width in human cancer cells.
f. Same as (e) but for mouse liver.
g. Fraction of down-regulated genes in genes with H3K4me3 peaks with sensitive width and all other genes with H3K4me3 in human cancer cells.
h. Number of significantly enriched (GSEA FDR Q-value<1e-5) pathways in down-regulated genes and up-regulated genes in human cancer cells.
i. Annotation of 305 pathways enriched in down-regulated genes in human cancer cells.
j. Fraction of up-regulated genes in genes with H3K4me3 peaks with robust width and all other genes with H3K4me3 in mouse liver.
k. Same as in (h) but for mouse liver.
l. Annotation of 65 pathways enriched in up-regulated genes in mouse liver.

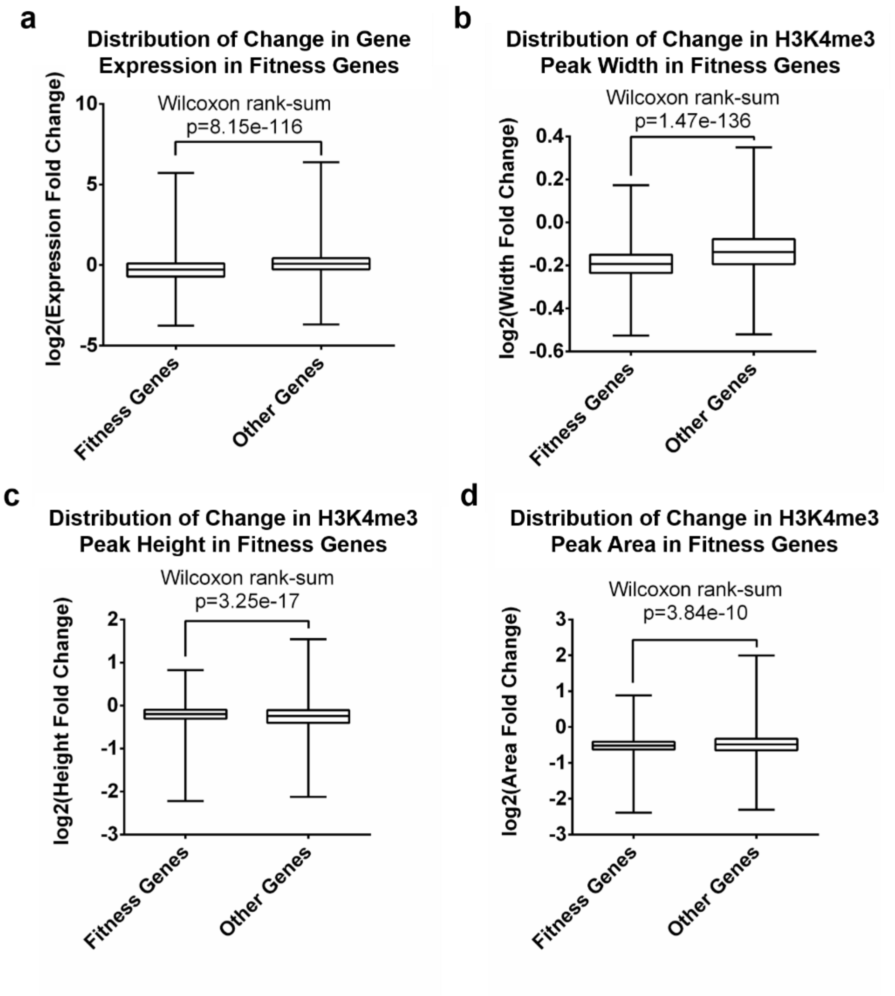

Supplementary Figure 12 (Related to Fig. 4). Fitness genes in human cancer cells are more sensitive to MR.

a. Distribution of gene expression changes in fitness genes (i.e. genes found to be essential for cancer cell survival and proliferation in a CRISPR-based screen) and all other genes. Box limits are the 25th and 75th percentiles, center lines are medians, and the whiskers are the minimal and maximal values.

b. Distribution of width changes in H3K4me3 peaks associated with fitness genes and all other genes. Box limits are the 25th and 75th percentiles, center lines are medians, and the whiskers are the minimal and maximal values.

c. Same as in (b) but for height.

d. Same as in (b) but for area.
