# Supplementary material for: Methionine metabolism influences genomic architecture and gene expression through H3K4me3 peak width
Source: Nat Commun. 2018 May 16;9:1955. doi: 10.1038/s41467-018-04426-y (PMC5955993; doi:10.1038/s41467-018-04426-y)
Supplement: Supplementary file 3 — Description of Additional Supplementary Files [file 41467_2018_4426_MOESM3_ESM.pdf]

Description of Additional Supplementary Files

File Name: Supplementary Data 1
Description: Peak height, area, width and gene expression levels in different conditions.
